# Is There Any Association between Use of Smokeless Tobacco Products and Coronary Heart Disease in Bangladesh?

**DOI:** 10.1371/journal.pone.0030584

**Published:** 2012-01-20

**Authors:** Muhammad Aziz Rahman, Nicola Spurrier, Mohammad Afzal Mahmood, Mahmudur Rahman, Sohel Reza Choudhury, Stephen Leeder

**Affiliations:** 1 Discipline of Public Health, The University of Adelaide, Adelaide, Australia; 2 Institute of Epidemiology, Disease Control and Research (IEDCR), Dhaka, Bangladesh; 3 National Heart Foundation Hospital & Research Institute (NHFH&RI), Dhaka, Bangladesh; 4 The Menzies Centre for Health Policy, The University of Sydney, Sydney, Australia; Universidad Peruana Cayetano Heredia, Peru

## Abstract

**Background:**

Most epidemiological studies exploring the association between smokeless tobacco (SLT) use and coronary heart disease (CHD) have been in Western populations, and have focused on SLT products used in those countries. Few studies come from South Asian countries. Our objective was to determine the association between SLT use and CHD among non-smoking adults in Bangladesh.

**Methods:**

A matched case-control study of non-smoking Bangladeshi adults aged 40–75 years was conducted in 2010. Incident cases of CHD were selected from two cardiac hospitals. Community controls, matched to CHD cases, were selected from neighbourhoods, and hospital controls were selected from outpatient departments of the same hospitals. The Rose Angina Questionnaire (RAQ) was also used to re-classify cases and controls.

**Results:**

The study enrolled 302 cases, 1,208 community controls and 302 hospital controls. Current use was higher among community controls (38%) compared to cases (33%) and hospital controls (32%). Current use of SLT was not significantly associated with an increased risk of CHD when community controls were used (adjusted OR 0.87, 95% CI 0.63–1.19), or when hospital controls were used (adjusted OR 1.00, 95% CI 0.63–1.60), or when both control groups were combined (adjusted OR 1.00, 95% CI 0.74–1.34). Risk of CHD did not increase with use of individual types except *gul*, frequency, duration, past use of SLT products, or using the RAQ to re-classify cases and controls. There was a significant association between *gul* use and CHD when both controls were combined (adjusted OR 2.93, 95% CI 1.28–6.70).

**Conclusions:**

There was no statistically significant association between SLT use in general and CHD among non-smoking adults in Bangladesh. Further research on the association between *gul* use and CHD in Bangladesh along with SLT use and CHD in other parts of the subcontinent will guide public health policy and interventions that focus on SLT-related diseases.

## Introduction

Smokeless tobacco (SLT), commonly used in many countries [1], is associated with various health effects. Epidemiological studies have consistently reported a significant positive association between SLT use and cancers of various organs such as oropharynx, oesophagus, stomach, pancreas, and lungs amongst others [2]. Studies also report a positive association between SLT use and oral diseases, dental diseases, hypertension, diabetes, poor reproductive outcomes, addiction, and all-cause mortality [2].

A number of studies have also reported a significant positive association between SLT use and risk factors for cardiovascular diseases (CVD) such as raised blood pressure and a less healthy lipid profile [3], [4]. However, the results of epidemiological studies assessing the association between SLT use and CHD, stroke or CVD in general are inconsistent [5], [6]. While several cohort [7], [8], [9] and case-control studies [10], [11] have reported a significant positive association, other sufficiently powered cohort [12], [13], [14] and case-control studies [15], [16], [17] have not reported such an association. Some studies undertaken in Western countries (Sweden and USA) have found an association [7], [8], [9] whereas others have not [12], [13], [14]. South Asian SLT products differ from Western products in terms of constituents, nicotine concentration, manufacturing, and storage methods [18]. Usage patterns are also likely to be different and may explain the different results from studies conducted across various settings [6], [19].

There are a limited number of studies from South Asian countries focusing on the association between SLT use and CHD. One Indian cohort study [20] and another multinational case-control study (INTERHEART) involving 52 countries [10] reported a significant positive association and did include South Asian SLT products. However, betel-quid and areca-nut were included as SLT products although these products do not contain tobacco. In addition, the INTERHEART study did not report results separately for any South Asian country [10]. A small number of Taiwanese studies [21], [22], [23] found a significant positive association between betel-quid chewing and CHD, but not with SLT use. The only study which has included SLT products available in Bangladesh [11], all of which contain tobacco, showed a significant positive association between SLT use and CHD (adjusted odds ratio 2.2, 95% confidence interval 1.1–4.5) and was conducted by the first author.

As a developing country in South-East Asia, Bangladesh has high rates of smoking and SLT usage. Half of those aged ≥15 years (43%≈41 million) use tobacco in some form [24]. The prevalence of SLT use has been estimated as 27% with similar rates in men (26%) and women (28%), but more prevalent in rural areas (29%) compared to urban areas (23%) [24]. Whilst a number of studies in Bangladesh have examined tobacco use [25], [26], [27], the only study focusing on the SLT-CHD association [11] had a small sample size (n = 207), included smokers, and recruited cases and controls from a hospital setting.

Betel-leaf (*paan*) chewing is a cultural tradition of Bangladeshi people extending back many centuries [28]. In Bangladesh, as in other countries of the subcontinent, people chew betel-leaf with/without SLT products routinely at various cultural and social events [29]. As there has been no large systematic study conducted in the Subcontinent, and the results of studies conducted in Western settings are inconsistent [6], we conducted the current study to determine whether there was any association between SLT use and CHD among non-smoking adults in Bangladesh.

## Methods

### Ethics statement

Informed written consent was requested from each participant in the prescribed consent form. Privacy and confidentiality were maintained regarding the collected data. The protocol including the information sheet and consent forms for this project was approved by The University of Adelaide Human Research Ethics Committee, Australia (H-117-2009) and the local ethics committee of Bangladesh Medical Research Council, Bangladesh (BMRC/NREC/2007–2010/125).

### Study design and study sites

A matched case-control study was conducted in 2010. Data were collected through structured interviews. CHD cases were recruited from inpatient facilities of the National Institute of Cardiovascular Diseases (NICVD) and the National Heart Foundation Hospital and Research Institute (NHFH&RI), Dhaka, Bangladesh. Both hospitals are accessible to people from all socio-economic groups as minimal costs are associated with cardiac care. During the recruitment period, approximately 550 patients per day were admitted to the six cardiovascular units of NICVD and 110 patients per day were admitted to the seven cardiovascular units of NHFH&RI. Four hundred patients per day and 75 patients per day attended the outpatient facilities of the NICVD and the NHFH&RI respectively. Both hospital controls and community controls were selected in this study in order to assess whether results differed according to the use of different control groups. Hospital controls were recruited amongst individuals attending cardiac outpatient facilities of the NICVD and the NHFH&RI, while community controls were recruited from the neighbourhood households of CHD cases within Dhaka City Corporation (DCC) areas.

### Study population

Inclusion criteria were: age 40–75 years, non-smoker, residence within DCC areas, and well enough to undertake a 20 minute interview. Non-smokers were defined as either (i) never smokers or (ii) ex-smokers who had not smoked a single puff in the past 10 years. This was because most studies suggest that the maximum reduction in CHD risk occurs within 4–14 years following smoking cessation [30], [31], [32]; and from a practical perspective, only including never smokers would have been difficult.

### CHD cases

CHD patients admitted to the two hospitals and diagnosed as incident cases of CHD (diagnosis for the first time within the preceding twelve months) by hospital cardiologists, were selected as cases. Cardiologists diagnosed CHD cases based on clinical judgment (a combination of classical symptoms with positive results from electrocardiogram, cardiac enzymes, exercise tolerance test, or coronary artery angiogram). Either angina and/or myocardial infarction were included in the definition of CHD for the purpose of this study.

### Community controls

Neighbourhood residents of the CHD cases, who had no self-reported cardiac disease, were selected as community controls. Control subjects were matched by age (±5 years), sex and socio-economic status (SES) to the corresponding case. If a suitable control subject could not be located in a suburb of the CHD case, the next adjacent suburb was used (this happened in 28% of cases).

### Hospital controls

Hospital controls were also used in this study. This was to determine whether any systematic bias existed in the use of hospital controls as is often postulated in the literature [33], [34]. These additional analyses are not the focus of this particular article and will be presented elsewhere. Patients, who attended cardiac outpatient facilities of the same hospitals and were diagnosed as not suffering from CHD by hospital cardiologists, were selected as hospital controls. It should be noted that unlike a developed country, many individuals with symptoms of chest pain or breathlessness attend outpatient facilities of cardiac hospitals for screening of cardiac disease; either self-referred or referred by a general practitioner in Bangladesh. About two-thirds (64%) of the hospital controls were selected from the hypertension clinic of NHFH&RI, which was the only available source of recruiting controls in that study setting. This poses a risk of potential bias because SLT use is known to be associated with hypertension [3]. Diagnoses for these patients included hypertension (62%), non-specific chest pain (48%), and gastric hyper-acidity (13%). Some patients were not assigned a diagnosis, and symptoms of palpitation (10%) or breathlessness (8%) were given in the case-notes. Each hospital control was matched with a corresponding case by age (±5 years) and sex.

### Cases and controls re-classified by the Rose Angina Questionnaire (RAQ)

**Figure 1:**
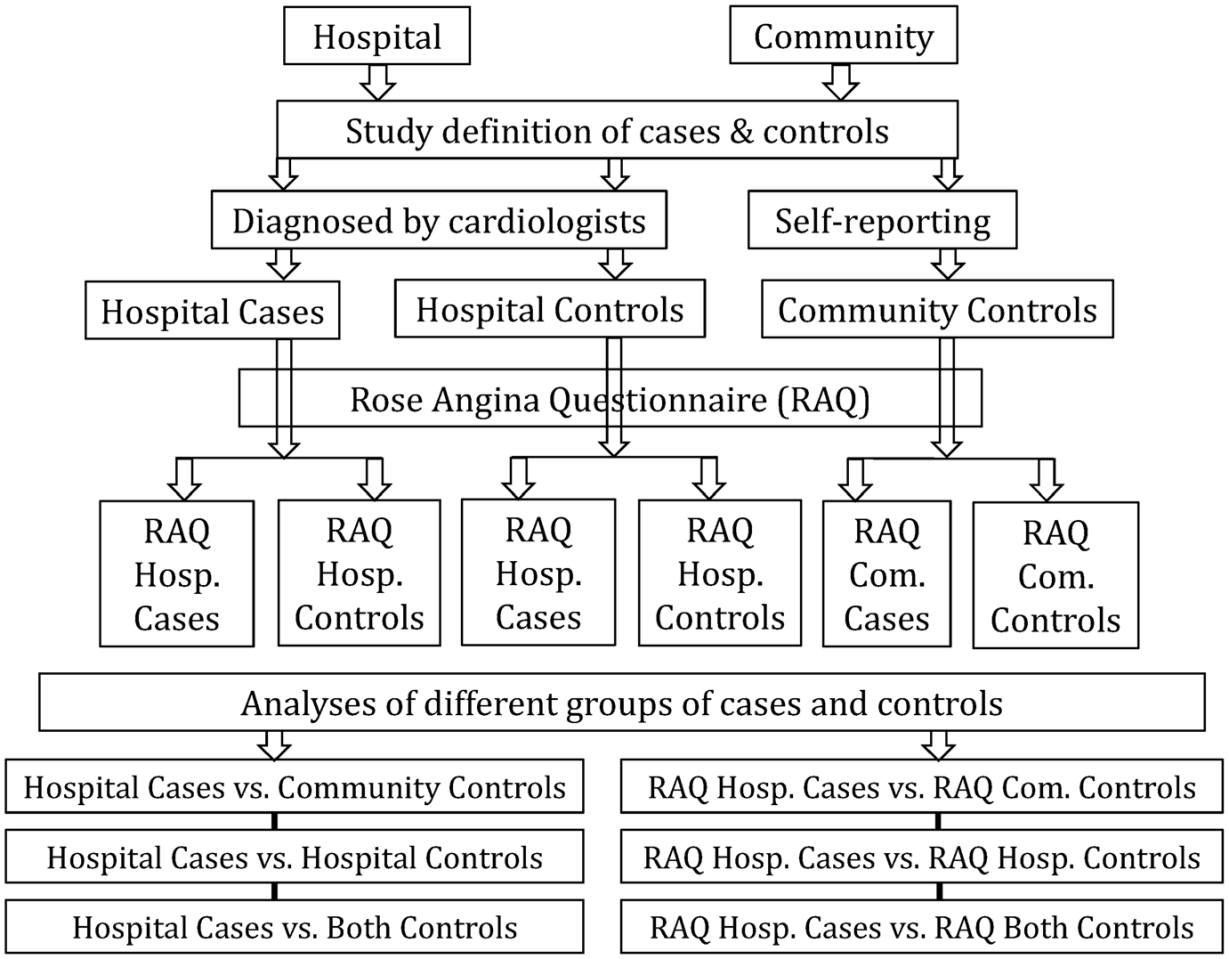
Re-classification of cases and controls using the Rose Angina Questionnaire (RAQ).

In addition to our study definition of cases and controls, we also used the RAQ [35] to re-classify study participants into RAQ cases and RAQ controls. Individuals responding affirmatively to the RAQ were re-classified as RAQ cases and the negative responders were re-classified as RAQ controls as shown in Figure 1. It is to be noted that the RAQ cases and the RAQ controls were not matched.

### Sample size

Sample size was calculated using Epi-info version 3.5.1. With 95% confidence intervals, 80% power, a control: case ratio of 4∶1, a correlation for matched design of 0.1, an expected frequency of SLT use among controls of 25% [25], and a clinically significant odds ratio considered to be 1.5 [11], [20], 302 cases and 1,208 controls were required for this study. Additionally, one hospital control was selected for each case (302 additional controls).

### Study tool

A structured interview was conducted to measure exposure and confounding variables. Initially, a screening questionnaire was used to select eligible cases and controls. This included information on age, residence, smoking and heart disease status. Once informed consent was obtained, participants were asked a range of questions covering socio-demographic information, a detailed history of SLT use, and other known risk factors for CHD. Socio-demographic information included age, gender, marital status, highest level of education achieved, primary occupation and monthly house-rent as a proxy to socio-economic status.

Betel-leaf or areca-nut alone was not included as a SLT product, as they do not contain tobacco. If a respondent used any SLT product with/without betel-leaf or areca-nut in the last twelve months, he/she was categorized as a current SLT user. If a respondent ceased using SLT products for at least last twelve months, he/she was categorized as a past SLT user. If a respondent was not using any SLT product currently or in the past, he/she was categorized as never a tobacco user (as they were also non-smokers according to the participant selection criteria). Using frequency and duration of SLT use, we categorized frequency into light use (less than once a day) and heavy use (at least once a day), duration into short duration (<10 years) and long duration (>10 years), and quit duration into short-term quit (2–10 years) and long-term quit (>10 years).

Information on known risk factors for CHD included self-reported history of hypertension, diabetes, family history of heart disease, level of physical activity, use of hormonal contraceptives for women, exposure to indoor passive smoking, and occurrence of acute psycho-social events within last one year.

### Data collection

Reasons for non-participation were documented. If participants asked whether SLT could cause any health effect, interviewers only provided this information at the completion of the interview. Categorization of CHD cases according to the case definition and the RAQ was undertaken by the first author and selection of the majority of controls was undertaken in his presence. The first author trained the interviewers and undertook regular supervision of all data collection activities. In addition, the first author re-interviewed 4 cases (1%), 24 community controls (2%) and 6 hospital controls (2%) as a means of quality control of data collection.

### Laboratory analysis

To enhance interpretation of the study results, samples of SLT products most commonly used within the DCC areas, were tested for nicotine. Purchased samples of *paan-masala* (3 samples), *jarda* (1 sample), and *gul* (1 sample) were analysed following extraction, steam distillation and silicotungstic acid gravimetric method [36] at the Institute of Food Science and Technology (IFST), Bangladesh Council of Scientific and Industrial Research (BCSIR). Nicotine concentration was reported in percentage by weight (% by wt.). *Sada-pata*, which is the natural tobacco leaf in dried form, was also used by study participants but was not tested for nicotine because of the natural variation of this product. A recent surveillance study reported nicotine concentrations in Bangladeshi *sada-pata* as 1.97% [37].

### Data analysis

Analyses were performed using STATA version 10 statistical software. Initially, categorical variables were described as proportions for socio-demographic variables, SLT use, and risk factors for CHD. To determine the association between SLT use and CHD, cases and controls were compared using cross-tabulations at first. To statistically compare cases and controls, we used McNemar's chi-squared (χ^2^) tests when the frequency in all of the cells of the cross-tabulation was ≥5 and Fisher's exact test otherwise. Univariate conditional logistic regression models [38] were fitted to determine the strength of the association between SLT use and CHD, with the effect of SLT use expressed as a matched odds ratios (ORs) with 95% confidence intervals (CIs). Then multivariate conditional logistic regression models were fitted to adjust for potential confounding variables. The most important confounder is the presence of hypertension; this is particular so with analysis using only hospital controls. Confounding variables were identified initially using a χ^2^ test relating the variables to CHD. If the p-value from the χ^2^ test was less than 0.20 and there was no missing data for the confounder, that variable was included into the final multivariate analysis. The adjusted ORs with 95% CIs finally determined the association between SLT use and CHD in this study. To determine whether the inclusion of ex-smokers could have biased the results, analyses were conducted separately for never-smokers, ex-smokers, and combining both groups. Data were analysed separately using community controls, hospital controls, and combining both control groups. We also analysed data with all groups of re-classified cases and controls done by the RAQ to further explore the association between SLT use and CHD (Figure 1). As the RAQ cases and the RAQ controls were not matched, we used univariate and multivariate logistic regression models for these analyses.

## Results

### Study participants

Eligible participants included 311 hospital cases, 1293 community controls and 316 hospital controls. Nine potential hospital cases (3%), 85 potential community controls (7%), and 14 potential hospital controls (4%) did not consent to participate. Thus, the overall response rate was 94%. Results for the remaining 302 CHD cases from two cardiac hospitals, 1208 community controls and 302 hospital controls are presented in this paper.

Mean age of participants was 53 years (standard deviation ±8.5 years), 49.7% were men. Table 1 shows the distribution of different socio-demographic variables among cases and controls, and there were no significant differences in socio-demographic variables comparing cases and controls. Amongst the 1812 participants, 1292 (71%) were never-smokers. Never-smoking status was similar between cases (203 out of 302, 67%) and either community controls (864 out of 1208, 72%) or hospital controls (225 out of 302, 75%).

**Table 1:** Socio-demographic and risk factor variables for coronary heart disease (CHD) among the study participants.

Socio-demographic variables	Total, N (%)	Cases, n(%)	Community Controls, n(%)	Hospital controls, n(%)
Total study participants	1812	302	1208	302
Age in years, mean (SD)	53.0 (±8.5)	53.5 (±8.5)	53.1 (±8.5)	51.9 (±8.4)
Male participants	900 (49.7)	150 (49.7)^a^	600 (49.7)^a^	150 (49.7)^a^
Married (and living with spouse)	1414 (78.0)	232 (76.8)^a^	939 (77.8)^a^	243 (80.5)^a^
Highest level of education achieved				
Illiterate	204 (11.3)	34 (11.3)^a^	151 (12.5)^a^	19 (6.3)^a^
Can sign names	212 (11.7)	27 (8.9)^a^	150 (12.4)^a^	35 (11.6)^a^
Primary	527 (29.1)	95 (31.5)^a^	338 (28.0)^a^	94 (31.2)^a^
Secondary	239 (13.2)	44 (14.6)^a^	153 (12.7)^a^	42 (14.0)^a^
Higher-secondary	197 (10.9)	33 (10.9)^a^	116 (9.6)^a^	48 (15.9)^a^
Above higher-secondary	418 (23.1)	66 (21.9)^a^	290 (24.0)^a^	62 (20.6)^a^
Primary occupation				
Service holder	558 (30.8)	87 (28.8)^a^	369 (30.6)^a^	102 (33.8)^a^
Businessmen	262 (14.5)	42 (13.9)^a^	180 (14.9)^a^	40 (13.2)^a^
Housewife	741 (40.9)	126 (41.7)^a^	495 (41.0)^a^	120 (39.7)^a^
Retired	235 (13.0)	47 (15.6)^a^	149 (12.3)^a^	39 (12.9)^a^
Socio-economic status (SES) by monthly house-rent (HR)				
Lower SES (HR<5000 BDT)	656 (36.2)	109 (36.1)^a^	433 (35.8)^a^	114 (37.7)^a^
Middle SES (HR 5000–10000 BDT)	930 (51.3)	152 (50.3)^a^	620 (51.3)^a^	158 (52.3)^a^
Higher SES (HR>10000 BDT)	226 (12.5)	41 (13.6)^a^	155 (12.8)^a^	30 (9.9)^a^
Presence of other risk factors for CHD				
Hypertension^a^	796 (43.9)	180 (59.6)^a^	413 (34.2)^b^	203 (67.2)^a^
Diabetes^b^	446 (24.6)	129 (42.7)^a^	244 (20.2)^b^	73 (24.2)^b^
Family history of heart disease	421 (23.2)	94 (31.5)^a^	248 (21.5)^b^	79 (27.2)^a^
Undertook physical activity^c^	1116 (61.6)	179 (59.5)^a^	788 (65.3)^b^	149 (50.2)^b^
Use of hormonal contraceptives	60 (3.3)	9 (3.0)^a^	41 (3.4)^a^	10 (3.3)^a^
Exposure to indoor passive smoking^d^	321 (17.7)	58 (19.2)^a^	218 (18.0)^a^	45 (14.9)^a^
Acute psycho-social stress^e^	434 (24.0)	94 (31.1)^a^	265 (21.9)^b^	75 (24.8)^a^

Superscripts indicate which categories show a statistically significant (p<0.05) difference using chi-squared tests between cases and controls: same letter indicates no difference, different letter indicates a difference.

a “Have you ever been told by a doctor or a health-worker that you have raised blood-pressure or hypertension?”

b “Have you ever been told by a doctor or a health-worker that you have raised blood-glucose or diabetes?”

c Physical activity included moderate to vigorous physical activity for at least 30 minutes per week which made them huff and puff (where they can still talk but can't sing). There were three levels of physical activity: mild (1–2 times/week), moderate (3–4 times/week) and vigorous (≥5 times/week). All these three levels were combined together in this table.

d “Does anyone smoke inside the same room, where you live?”

e Such an incident that caused mental agony, sorrow, unhappiness or anxiety within last one year, like death of family members, divorce, separation, sudden job loss, unemployment, financial loss etc.

### Risk factors for CHD

Table 1 shows the distribution of risk factors for CHD among cases and controls. More than two-thirds of hospital controls (67%) were hypertensive compared to half of cases (60%) and one-third of community controls (34%).The majority of these hospital controls were selected from the hypertension clinic of one study hospital which explains this difference.

### Nicotine content of the SLT products

Nicotine was absent in all three commercial samples of *paan-masala* products tested. The selected samples of *jarda* and *gul* contained 0.96% and 5.48% nicotine respectively. Therefore, our data analysis included only three types of SLT products containing nicotine: *jarda*, *sada-pata* (1.97% nicotine) and *gul*.

### Use of SLT products

Amongst the 1812 participants, 648 (36%) were current SLT users. Current use was higher among community controls (38%) compared to that of cases (33%) and hospital controls (32%). Quitting was more common among cases compared to either group of controls. Amongst the never-smoker participants, current use of SLT was more common among community controls (35%) than that of cases (25%) and hospital controls (30%). Amongst the ex-smoker participants, ever use, current use and quitting of SLT products were more common among cases compared to either group of controls. Table 2 shows the status of SLT use among the study participants.

**Table 2:** Univariate and multivariate matched analysis showing the association between coronary heart disease and use of smokeless tobacco (by smoking status of the participants).

	Hospital cases vs. community controls						Hospital cases vs. hospital controls						Hospital cases vs. both controls					
	Hospital Cases, n(%)	Community Controls, n(%)	OR	95% CI	Adj. OR§	95% CI	Hospital Cases, n(%)	Hospital Controls, n(%)	OR	95% CI	Adj. OR*	95% CI	Hospital Cases, n(%)	Both Controls, n(%)	OR	95% CI	Adj. OR^¶^	95% CI
Never users of any tobacco (Reference)			1.00		1.00				1.00		1.00				1.00		1.00	
Non-smokers (total study participants)	302	1208					302	302					302	1510				
Ever users of SLT products	118 (39.1)	482 (39.9)	0.98	0.74–1.29	0.96	0.71–1.30	118 (39.1)	101 (33.4)	1.33	0.94–1.87	1.11	0.71–1.71	118 (39.1)	583 (38.6)	1.04	0.80–1.37	1.08	0.81–1.44
Current users of SLT products	99 (32.8)	454 (37.6)	0.87	0.65–1.17	0.87	0.63–1.19	99 (32.8)	95 (31.5)	1.18	0.83–1.69	1.00	0.63–1.60	99 (32.8)	549 (36.4)	0.93	0.70–1.24	1.00	0.74–1.34
Quitters of SLT products	19 (6.3)	28 (2.3)	2.38	1.16–4.91	2.04	0.92–4.57	19 (6.3)	6 (2.0)	4.00	1.13–14.2	2.19	0.51–9.44	19 (6.3)	34 (2.3)	2.57	1.30–5.11	2.08	0.99–4.35
Never-smokers	203	864					203	225					203	1089				
Ever users of SLT products	60 (29.6)	313 (36.2)	0.71	0.49–1.02	0.66	0.44–0.99	60 (29.6)	71 (31.6)	0.92	0.59–1.45	1.11	0.59–2.10	60 (29.6)	384 (35.3)	0.76	0.53–1.07	0.77	0.52–1.13
Current users of SLT products	51 (25.1)	299 (34.6)	0.65	0.44–0.95	0.62	0.40–0.95	51 (25.1)	68 (30.2)	0.86	0.54–1.39	1.19	0.62–2.31	51 (25.1)	367 (33.7)	0.70	0.48–1.01	0.76	0.51–1.13
Quitters of SLT products	9 (4.4)	14 (1.6)	1.49	0.53–4.24	1.09	0.35–3.40	9 (4.4)	3 (1.3)	2.00	0.37–10.9	0.37	0.02–5.48	9 (4.4)	17 (1.6)	1.46	0.54–3.94	0.97	0.33–2.85
Ex-smokers	99	344					99	77					99	421				
Ever users of SLT products	58 (58.6)	169 (49.1)	1.48	0.84–2.59	1.52	0.83–2.79	58 (58.6)	30 (39.0)	2.80	1.01–7.78	2.12	0.63–7.18	58 (58.6)	199 (47.3)	1.58	0.92–2.72	1.44	0.82–2.53
Current users of SLT products	48 (48.5)	155 (45.1)	1.54	0.84–2.83	1.50	0.78–2.89	48 (48.5)	27 (35.1)	2.20	0.76–6.33	0.85	0.20–3.55	48 (48.5)	182 (43.2)	1.61	0.89–2.90	1.42	0.77–2.63
Quitters of SLT products	10 (10.1)	14 (4.1)	1.03	0.30–3.60	0.91	0.15–5.49	10 (10.1)	3 (3.9)	Undef	Undef	Undef	Undef	10 (10.1)	17 (4.0)	1.51	0.46–4.99	1.57	0.34–7.17

The variables those were significant (p<0.20) during initial univariate analysis, were selected as confounders and adjusted during multivariate analysis.

§Adjusted for: age, hypertension, diabetes and acute psycho-social stress; matched for: age, sex, residential areas and monthly house rent.

*Adjusted for: age, marital status, hypertension, diabetes, indoor smoking exposure and acute psycho-social stress; matched for: age and sex.

¶ Adjusted for: age, hypertension, diabetes and acute psycho-social stress; matched for: age, sex, residential areas and monthly house rent.

Amongst the individual types of SLT products, use of *jarda* was more common compared to *sada-pata* and *gul*. Current use of *jarda* was slightly higher among community controls (26%) compared to either cases (21%) or hospital controls (24%). There was no difference between cases and controls for current use of *sada-pata*. Current use of *gul* was slightly more common among cases (5%) compared to either group of controls (2%). The majority of exclusive *jarda*, *sada-pata* or *gul* consumers were heavy users and long duration users. Mean duration of *jarda* use was 16 years (0.1–55 years), *sada-pata* 28 years (3–60 years), and *gul* 17 years (0.5–45 years). There was no difference between cases and controls for heavy use or long duration use of each SLT product.

### Association between SLT use and CHD

Table 2, 3, 4 show the results of univariate and multivariate analyses. Among the socio-demographic variables and risk factor variables for CHD, age, hypertension, diabetes, and acute psycho-social stress were significantly associated with CHD when data were analysed using community controls, hospital controls or both controls. In addition, marital status and indoor passive smoking were significantly associated with CHD when data were analysed with hospital controls. There was no statistically significant association between current SLT use and CHD when community controls were used (adjusted OR 0.87, 95% CI 0.63–1.19), or hospital controls were used (adjusted OR 1.00, 95% CI 0.63–1.60), or when both controls were combined (adjusted OR 1.00, 95% CI 0.74–1.34). There was no association between ever use or cessation of SLT usage and CHD. Similar results were found when data were analysed separately for never-smokers and ex-smokers. Similarly, Table 3 shows that there was no statistically significant association between SLT use and CHD, when data were analysed using the RAQ classified cases and RAQ classified controls.

**Table 3:** Univariate and multivariate unmatched analysis showing the association between coronary heart disease and use of smokeless tobacco among the re-classified cases and controls by the Rose Angina Questionnaire (RAQ).

	RAQ hospital cases vs. RAQ community controls						RAQ hospital cases vs. RAQ hospital controls						RAQ hospital cases vs. RAQ both controls					
	RAQ Hospital Cases, n(%)	RAQ Community Controls, n(%)	OR	95% CI	Adj. OR §	95% CI	RAQ Hospital Cases, n(%)	RAQ Hospital Controls, n(%)	OR	95% CI	Adj. OR *	95% CI	RAQ Hospital Cases, n(%)	RAQ Both Controls, n(%)	OR	95% CI	Adj. OR ¶	95% CI
Never users of any tobacco (Reference)			1.00		1.00				1.00		1.00				1.00		1.00	
Non-smokers (total study participants)	194	1153					194	409					194	1562				
Ever users of SLT products	78 (40.2)	458 (39.7)	1.02	0.75–1.39	0.94	0.66–1.34	78 (40.2)	143 (35.0)	1.25	0.88–1.78	1.08	0.72–1.61	78 (40.2)	601 (38.5)	1.08	0.79–1.46	1.02	0.73–1.43
Current users of SLT products	69 (35.6)	431 (37.4)	0.96	0.70–1.32	0.88	0.61–1.27	69 (35.6)	129 (31.5)	1.23	0.85–1.77	1.08	0.72–1.63	69 (35.6)	560 (35.9)	1.02	0.74–1.40	0.98	0.70–1.39
Quitters of SLT products	9 (4.6)	27 (2.3)	2.00	0.92–4.35	1.96	0.83–4.63	9 (4.6)	14 (3.4)	1.47	0.62–3.50	1.04	0.38–2.85	9 (4.6)	41 (2.6)	1.82	0.86–3.84	1.65	0.74–3.68
Never-smokers	132	823					132	295					132	1118				
Ever users of SLT products	42 (31.8)	296 (36.0)	0.83	0.56–1.23	0.76	0.48–1.19	42 (31.8)	90 (30.5)	1.06	0.68–1.65	0.82	0.49–1.39	42 (31.8)	386 (34.5)	0.88	0.60–1.30	0.85	0.55–1.30
Current users of SLT products	37 (28.0)	282 (34.3)	0.77	0.51–1.16	0.71	0.45–1.13	37 (28.0)	85 (28.8)	0.99	0.63–1.57	0.79	0.46–1.35	37 (28.0)	367 (32.8)	0.82	0.55–1.23	0.80	0.51–1.24
Quitters of SLT products	5 (3.8)	14 (1.7)	2.09	0.74–5.95	1.98	0.62–6.29	5 (3.8)	5 (1.7)	2.28	0.64–8.06	1.82	0.39–8.53	5 (3.8)	19 (1.7)	2.14	0.78–5.87	2.02	0.68–6.03
Ex-smokers	62	330					62	114					62	444				
Ever users of SLT products	36 (58.1)	162 (49.1)	1.44	0.83–2.49	1.34	0.71–2.53	36 (58.1)	53 (46.5)	1.59	0.85–2.98	1.12	0.54–2.36	36 (58.1)	215 (48.4)	1.47	0.86–2.52	1.35	0.75–2.42
Current users of SLT products	32 (51.6)	149 (45.2)	1.39	0.79–2.44	1.25	0.65–2.43	32 (51.6)	44 (38.6)	1.71	0.89–3.26	1.22	0.56–2.66	32 (51.6)	193 (43.5)	1.46	0.84–2.54	1.35	0.73–2.48
Quitters of SLT products	4 (6.5)	13 (3.9)	1.99	0.60–6.56	2.19	0.52–9.19	4 (6.5)	9 (7.9)	1.04	0.29–3.69	0.73	0.16–3.34	4 (6.5)	22 (5.0)	1.60	0.51–5.01	1.41	0.40–5.04

The variables those were significant (p<0.20) during initial univariate analysis, were selected as confounders and adjusted during multivariate analysis.

§Adjusted for: age, socioeconomic status, hypertension, diabetes, family history of heart disease, physical activities, indoor smoking and acute psycho-social stress.

*Adjusted for: marriage, level of education, socioeconomic status, hypertension, diabetes, indoor smoking and acute psycho-social stress.

¶Adjusted for: age, socioeconomic status, hypertension, diabetes, family history of heart disease, indoor smoking and acute psycho-social stress.

**Table 4:** Univariate and multivariate matched analysis showing the association between coronary heart disease and current use of different types of Bangladeshi smokeless tobacco (SLT) products (by smoking status of the participants).

	Hospital cases vs. community controls						Hospital cases vs. hospital controls						Hospital cases vs. both controls					
	Hospital Cases, n(%)	Community Controls, n(%)	OR	95% CI	Adj. OR§	95% CI	Hospital Cases, n(%)	Hospital Controls, n(%)	OR	95% CI	Adj. OR*	95% CI	Hospital Cases, n(%)	Both Controls, n(%)	OR	95% CI	Adj. OR¶	95% CI
Never users of any tobacco (Reference)			1.00		1.00				1.00		1.00				1.00		1.00	
Non-smokers (total study participants)	302	1208					302	302					302	1510				
Current users of any one SLT product																		
*Jarda* (0.96% nicotine)	63 (20.9)	314 (26.0)	0.75	0.53–1.06	0.79	0.55–1.15	63 (20.9)	71 (23.5)	0.86	0.56–1.33	0.72	0.40–1.28	63 (20.9)	385 (25.5)	0.79	0.56–1.10	0.84	0.59–1.18
*Sada-pata* (1.97% nicotine)	8 (2.6)	28 (2.3)	1.17	0.47–2.93	1.14	0.41–3.22	8 (2.6)	8 (2.6)	1.25	0.34–4.65	1.39	0.29–6.65	8 (2.6)	36 (2.4)	1.17	0.49–2.79	1.26	0.50–3.20
*Gul* (5.48% nicotine)	15 (5.0)	28 (2.3)	2.70	1.11–6.54	2.23	0.87–5.70	15 (5.0)	6 (2.0)	3.67	1.02–13.1	2.84	0.58–13.9	15 (5.0)	34 (2.3)	2.82	1.27–6.25	2.93	1.28–6.70
Never-smokers	203	864					203	225					203	1089				
Current users of any one SLT product																		
*Jarda* (0.96% nicotine)	34 (16.7)	205 (23.7)	0.60	0.38–0.94	0.59	0.36–0.96	34 (16.7)	52 (23.1)	0.66	0.37–1.17	0.89	0.39–2.00	34 (16.7)	257 (23.6)	0.60	0.39–0.93	0.64	0.40–1.02
*Sada-pata* (1.97% nicotine)	5 (2.5)	21 (2.4)	0.95	0.34–2.68	0.90	0.27–2.98	5 (2.5)	8 (3.6)	1.25	0.34–4.65	1.66	0.32–8.64	5 (2.5)	29 (2.7)	0.98	0.37–2.59	1.11	0.39–3.18
*Gul* (5.48% nicotine)	7 (3.4)	18 (2.1)	1.44	0.37–5.55	1.33	0.31–5.69	7 (3.4)	3 (1.3)	2.00	0.37–10.9	0.82	0.07–9.09	7 (3.4)	21 (1.9)	1.56	0.47–5.18	1.42	0.38–5.24
Ex-smokers	99	344					99	77					99	421				
Current users of any one SLT product																		
*Jarda* (0.96% nicotine)	29 (29.3)	109 (31.7)	1.44	0.71–2.90	1.51	0.71–3.24	29 (29.3)	19 (24.7)	2.00	0.50–8.00	0.56	0.08–3.82	29 (29.3)	128 (30.4)	1.48	0.76–2.89	1.52	0.76–3.05

The variables those were significant (p<0.20) during initial univariate analysis, were selected as confounders and adjusted during multivariate analysis.

§Adjusted for: age, hypertension, diabetes and acute psycho-social stress; matched for: age, sex, residential areas and monthly house rent.

*Adjusted for: age, marital status, hypertension, diabetes, indoor smoking exposure and acute psycho-social stress; matched for: age and sex.

¶Adjusted for: age, hypertension, diabetes and acute psycho-social stress; matched for: age, sex, residential areas and monthly house rent.

When we stratified our analyses according to younger (40–57 years) and older (58–75 years) age groups, there was no statistically significant association between current SLT use and CHD among younger and older participants, when community controls were used (younger: adjusted OR 1.08, 95% CI 0.73–1.60, older: adjusted OR 0.54, 95% CI 0.27–1.07), or hospital controls were used (younger: adjusted OR 1.14, 95% CI 0.59–2.19, older: adjusted OR 0.89, 95% CI 0.32–2.47), or when both controls were combined (younger: adjusted OR 1.19, 95% CI 0.82–1.72, older: adjusted OR 0.75, 95% CI 0.42–1.32). Results did not change when data were analysed separately for never-smokers and ex-smokers.

When we stratified our analyses further according to gender, there was no statistically significant association between current SLT use and CHD among men and women, when community controls were used (men: adjusted OR 1.30, 95% CI 0.81–2.10, women: adjusted OR 0.62, 95% CI 0.38–0.99), or hospital controls were used (men: adjusted OR 1.08, 95% CI 0.46–2.55, women: adjusted OR 0.84, 95% CI 0.42–1.68), or when both controls were combined (men: adjusted OR 1.36, 95% CI 0.88–2.09, women: adjusted OR 0.73, 95% CI 0.47–1.14). Results did not change when data were analysed separately for never-smokers and ex-smokers.

Table 4 shows that there was no statistically significant association between use of *jarda* or *sada-pata* and CHD for current use, quitting or ever use during analyses by different control groups or by different smoking status. However, the product containing highest amount of nicotine (5.48%) in this study, *gul*, showed a significant positive association with CHD (adjusted OR 2.93, 95% CI 1.28–6.70), when data were analysed using both groups of controls.

There was no statistically significant association between frequency or duration of each SLT product use and CHD, except use of *gul.* There was a significant positive association between heavy use of *gul* and CHD (adjusted OR 2.78, 95% CI 1.17–6.57), and long duration use of *gul* and CHD (adjusted OR 3.57, 95% CI 1.26–10.1) when both controls were used. There may have been a problem with lack of power to make stratified analyses with each SLT product to identify the association with CHD, as there were very few users of each SLT product in this study (Table 4).

## Discussion

In this study, there was no statistically significant association between SLT use in general and CHD among non-smoking adults in Bangladesh. However, there was a significant association between use of *gul* and CHD. This is very important because whilst in general our study did not find an association between SLT use and CHD, if nicotine content is higher in SLT (as it is in some other countries), it is likely to pose a significant risk for the development of CHD. No significant association was found for frequency or duration of each SLT product except *gul*. Heavy use and long duration of *gul* use was significantly associated with CHD. Results did not change when community controls, hospital controls, or both control groups were used during analyses, and when never-smoker, ex-smoker, or both groups were used. The results were the same for current users, quitters or ever users of SLT products. In addition, re-classification of cases and controls utilizing the RAQ did not change the findings of association between SLT use and CHD. Separate analyses with different age groups and gender did not change the results as well.

Findings of this study are supported by earlier case-control [15], [16], [17], [39], cross-sectional [40] as well as cohort studies [12], [13], [14], [41], [42]. None of these case-control studies conducted in Sweden reported a statistically significant positive association between use of snuff and CHD, although the findings were for men only. Similar to case-control studies, none of these cohort studies have reported a significant association between SLT use and CHD. All of these cohort studies except the US study [12] included men only. The US study [12], which considered only fatal CHD, showed the same results when analysed separately for men (adjusted hazard ratio 0.6, 95% CI 0.3–1.2) and women (adjusted OR 1.4, 95% CI 0.8–2.2).

On the other hand, findings of this study are not supported by other cohort [7], [8], [9], [20] and case-control studies [10], [11]. The Swedish Construction Worker study [7] and the US Cancer Prevention Study [8] involving a larger cohort reported a significant positive association between SLT use and CHD. However, it is to be noted that both of these studies included men and fatal CHD only. Another US cohort study [9], which included both sexes as well as fatal and non-fatal CHD, reported a significant positive association between SLT use and CVD, but no separate results were reported for fatal and non-fatal CVD, or for CHD and stroke. All of these cohort studies included Western SLT products and populations. The only South Asian cohort study, conducted in India [20], showed a significant positive association between use of Indian SLT products and CHD among women (adjusted risk ratio 1.25, 95% CI 1.05–1.49), but not among men (adjusted risk ratio 0.89, 95% CI 0.75–1.05). This is also in contrast to what we have found in this study. The constituents of Indian SLT products are likely to be different from Bangladeshi SLT products, which could have resulted in the significant positive association in the Indian study. The INTERHEART [10] and the Bangladeshi case-control study [11] showed a significant positive association between SLT use and non-fatal CHD. All of these studies were limited by various methodological issues as described in the introduction to this paper and elsewhere [6].

The literature suggests inconsistent evidence regarding the association between SLT use and CHD among different age-groups. We did not find any difference in results by age, which is supported by another study that did not find any significant association among younger (35–54 years) and older (55–64 years) people [15]. On the other hand, whilst a cross-sectional study of Swedish construction workers did not find a significant association among younger workers (46–55 years) [40], the subsequent cohort study reported a significant association among young (35–54 years) as well as older workers (55–65 years) [7].

There was a significant association between use of *gul* and CHD in this study, although the numbers were not large enough to confirm this association from this study as mentioned before. *Gul* is the mixture of tobacco powder, molasses, alkaline modifiers and other ingredients prepared commercially, and used in other parts of South Asia including Bangladesh [1], [37]. This product is kept between cheek and gum, used alone unlike other SLT products which are usually used with betel leaf in Bangladesh. This product was reported as having the highest nicotine concentration in this study. A recent survey of SLT products from different countries also reported higher nicotine concentration in Bangladeshi *gul* compared to other SLT products [37]. Frequency and duration of *gul* use was also significantly associated with CHD in the present study. Further well-powered study need to explore the association between this specific SLT product and CHD in a more detailed way.

Results from the existing research in Western countries are inconclusive; studies from South Asia are very limited and have some methodological constraints [6]. The current study addressed some of these methodological issues. Strengths of this study comprise including only non-smoking participants, a wider age range, both men and women, both community controls and hospital controls, and including exclusive SLT products from Bangladesh. Inclusion of non-smokers controlled for the potential strong confounding effects of smoking on CHD at the design stage. In addition, potential confounders were measured and adjusted for. This was particularly important for hypertension, which had the potential of introducing bias when data were analysed using hospital controls. Increasing the age limit of the participants in contrast to the earlier Bangladeshi case-control study helped assess the association between SLT use and CHD among a broader and more representative sample of Bangladeshi population. The consistent findings regardless of using either hospital controls or community controls support the accuracy of the study results. For the exposure variable, betel-quid or areca-nut was not included as a SLT product unlike other prior studies; rather selection of SLT products was supported by direct analysis of nicotine content. Selecting subjects from the two tertiary care cardiac hospitals and the catchment areas within the DCC, which include people from all socio-economic strata, suggest our results are representative for urban dwellers in Bangladesh. However, the issue of different health care seeking behaviours should be kept in mind. Re-interviewing a percentage of both cases and controls ensured the quality of the collected data. Re-analysing data using the RAQ classification strengthened the study findings because milder or as yet undiagnosed CHD were identified from both hospitals and communities in this study.

The lack of an association between SLT use in general and CHD in this study can be explained in several ways. Nicotine concentration of some Bangladeshi SLT products, specifically *gul* is higher compared to commercial cigarettes (1.63%) or *bidi* smoking (2.12%) [43]. But more gradual and least peaked dosing of nicotine occurs for SLT use, although the blood concentration of nicotine remains similar for a daily SLT user and a smoker [44]. On the other hand, rapid dosing of nicotine occurs with smoking and this has the potential to result in much more intense cardiovascular stimulation [45]. Finally, SLT products do not contain carbon monoxide and polycyclic aromatic hydrocarbons, which are known to contribute to the cardiovascular effects of smoking [15], [45]. A significant association between *gul* use and CHD may be due to the higher nicotine concentration in the product itself along with the rapid absorption from buccal cavity to cause cardiovascular effects. It may also be due to other additives in *gul* having cardiovascular effects. Further studies need to confirm these hypotheses.

It was beyond the scope of this study to verify the self-reported diagnosis of non-CHD among the community controls by a qualified physician. Fatal CHD cases were not included in this study, because hospital death registers in Bangladesh are not well developed. In addition, collection of SLT exposure data from family members of deceased individuals would be less reliable compared to data collected from the users themselves. Reporting of the stratified analyses with each SLT product in this study has the potential to be biased as we had relatively small number of specific SLT users. We could not measure the amount of different SLT use from the study participants, as there are no standard pack sizes unlike snus or snuff. This limited us from including the amount during calculation of dose-response relationship between SLT use and CHD. However, as there was no association between SLT use and CHD, this missing information did not affect the result of this study. There is a chance of having interviewer-bias in this study, which can happen to any epidemiological study. But we had a structured questionnaire and the interviewers were trained to ask the exact question only, not try and interpret the questions for the respondents. However, as the interviewers could not be blinded, it is difficult to completely overcome this. Since our subjects were recruited from within Dhaka, our results may not be generalizable to the rural areas of Bangladesh. We attempted to measure and adjust for as many possible confounding variables as possible. Importantly, this included hypertension as previously discussed. However, it was not possible to measure body mass index (BMI) because we felt that urban dwellers in Bangladesh would be unlikely to invite interviewers into their homes to undertake height and weight measurements. Also, Bangladeshis do not tend to measure their own weight on a regular basis and so self-report data was also not considered feasible.

This study has implications for tobacco control policy. There is an ongoing debate regarding the use of SLT products as a safer alternative to active smoking and as a possible mechanism to encourage smoking cessation [2]. On the other hand, there is a concern that SLT use may potentiate tobacco smoking [46]. As tobacco control policies vary strikingly between countries [47], there is the potential of introducing Western SLT products as a harm-reduction agent into developing countries of South Asia [48]. Such products may contain ingredients, which could have unknown deleterious effects on CHD and other health conditions. In addition, SLT products and nicotine concentration also differ in other South Asian countries such as in India or Pakistan [1], [49].

This study did not find an association between SLT use in general and CHD among non-smoking Bangladeshi adults. This is the first large scale case-control study assessing the association between SLT use and CHD from a South Asian perspective. Despite the fact that the current study did not find an association between different Bangladeshi SLT products and CHD except *gul*, SLT use has an established risk for development of cancers and of dental diseases. Tobacco control campaigns should focus on these SLT-related diseases. Given the fact that the burden of tobacco-related illnesses are more among people of lower socio-economic status [25], as well as limited resources for health promotion activities in developing countries, policies supporting non-use of any form of tobacco are justified. Further research on the association between *gul* use and CHD in Bangladesh, along with SLT use and CHD in other parts of the subcontinent where SLT products may differ will guide public health policy and interventions to prevent SLT-related diseases. Because SLT use is not harmless, the strategic focus should be upon controlling both smoking and SLT use in Bangladesh.
